# Supplementary material for: Identification of photoperiod-induced specific miRNAs in the adrenal glands of Sunite sheep (Ovis aries)
Source: Front Vet Sci. 2022 Jul 22;9:888207. doi: 10.3389/fvets.2022.888207 (PMC9354845; doi:10.3389/fvets.2022.888207)
Supplement: Supplementary file 1 [file Data_Sheet_1.ZIP › Supplementary file/miRDeep2 file.zip!/miRDeep2 file/Novel_Structure/SP42A2/Novel_627.pdf]

| | |
|---|---|
| Provisional ID | : NC_019469.2_25174 |
| Score total | : 2.2 |
| Score for star read(s) | : -1.3 |
| Score for read counts | : 0 |
| Score for mfe | : 1.9 |
| Score for randfold | : 1.6 |
| Score for cons. seed | : |
| Total read count | : 34 |
| Mature read count | : 32 |
| Loop read count | : 2 |
| Star read count | : 0 |

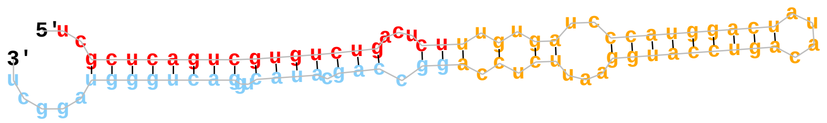


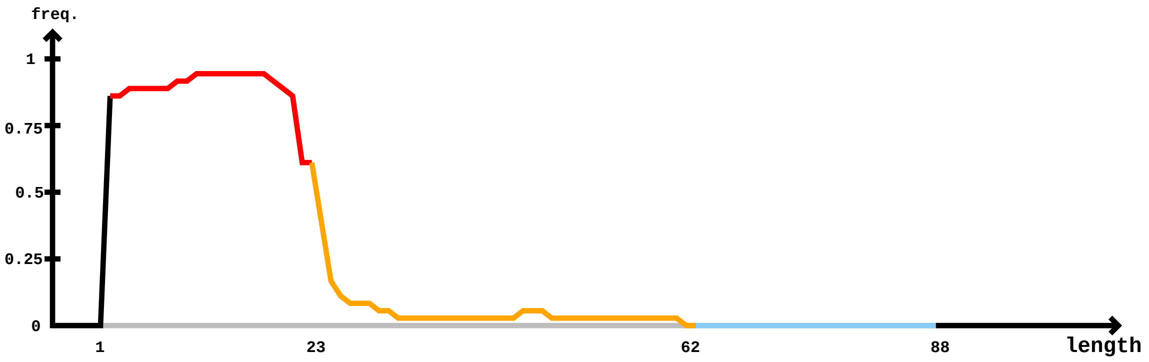


**Mature** **Star**

```
5'- aaagucgcucagucgugucugacucuuugugaucccauggacuauacaguccauggaauucuccaggccagcauacuggacuggguaggcuuucccuucccagggauc   -3'   exp
    .((((((((((((((((((...(((.(.((..((((((((....))))))))...)).).))).))).)))).))))))))).)))))(((((.....)))))..     reads   mm   sample
    ....ucgcucagucgugucug.......................................................................................   1   0   seq
    ....ucgcucagucgugucuga......................................................................................   1   0   seq
    ....ucgcucGgucgugucugac.....................................................................................   1   1   seq
    ....ucgcucGgucgugucugacu....................................................................................   5   1   seq
    ....ucgcucagucgugucugaUu....................................................................................   3   1   seq
    ....ucgcucagucgugucugacA....................................................................................   1   1   seq
    ....ucgcucagucgugucugacuc...................................................................................   1   0   seq
    ....ucgcucagucgugucugacucC..................................................................................   6   1   seq
    ....ucgAucagucgugucugacucu..................................................................................   2   1   seq
    ....ucgcucagucgugucugacucG..................................................................................   2   1   seq
    ....ucgcucagucgugucugacucA..................................................................................   4   1   seq
    ....ucgcucagucgugucugacucuA.................................................................................   2   1   seq
    ....ucgcucagucgugucugacucuAu................................................................................   1   1   seq
    ....ucUcucagucgugucugacucuuug...............................................................................   1   1   seq
    ......gcucagucgugucugacucuuA................................................................................   1   1   seq
    ..........Uucgugucugacucuuuguga.............................................................................   1   1   seq
    ............cgugucugacucuuugugauA...........................................................................   1   1   seq
    ........................uuugugaCcccauggacuauacagu...........................................................   1   1   seq
    ............................................aguccauggaauucucc...............................................   1   0   seq
```
